# Recent Advances in Electromagnetic Devices: Design and Optimization

**DOI:** 10.3390/mi16010098

**Published:** 2025-01-16

**Authors:** Chanik Kang, Haejun Chung

**Affiliations:** 1 Department of Artificial Intelligence, Hanyang University, Seoul 04763, Republic of Korea; chanik@hanyang.ac.kr; 2 Department of Electronic Engineering, Hanyang University, Seoul 04763, Republic of Korea

Electromagnetic devices are a continuous driving force in cutting-edge research and technology, finding applications in diverse fields such as optics [1,2,3], photonics [4], RF waves [5], and many others [6,7,8]. The design and optimization of electromagnetic devices have become essential to meet the demanding performance requirements for high efficiency, high power density, and reduced form factors. Over the years, various methods, such as analytic designs [9], evolutionary-based designs [10], gradient-based optimization [11,12], and neural network-based design techniques [13,14], have been employed to improve the design of electromagnetic devices.

Underpinning these design and optimization strategies are the fundamental principles of optics, photonics, and electromagnetics, which collectively drive modern technological advancements across a broad spectrum of applications. The field of optics focuses on the behavior and manipulation of light, including reflection, refraction, and diffraction, while photonics focuses on generating, controlling, and detecting photons for use in devices such as lasers, optical fibers, and imaging systems. Electromagnetics, the overarching discipline that unifies electric and magnetic phenomena, provides a theoretical framework for understanding how electromagnetic waves propagate and interact with various materials. Together, these fields enable innovations in high-speed communication systems [15], optical computing [16], and photonic integrated circuits [17], among others [18,19]. Continued research into advanced materials [20], fabrication processes [21], and simulation techniques [22,23,24] drives further progress, leading to the development of more compact, energy-efficient, and high-performance devices that continue to push the boundaries of what is possible in both fundamental science and practical applications.

This Special Issue features a diverse collection of recent advances in electromagnetic devices, offering insights and practical approaches that will be of significant value to researchers.

Zhang et al. [25] introduced a multifunctional metasurface designed for full-space electromagnetic wavefront control, which holds promise for applications in 6G communications. This work exemplifies how reconfigurability and compact designs can meet the growing demands for versatile electromagnetic systems. The authors achieved polarization conversion and reflection-beam pattern tuning, demonstrating a robust combination of theoretical modeling, simulation, and experimental validation.

Ma et al. [26] presented a 60 GHz slotted array horn antenna optimized for radar sensing in industrial scenarios. This work demonstrates the potential of millimeter-wave technologies in next-generation radar systems by achieving a high gain and wide bandwidth. Their method involved meticulous radiation-band structure design and array optimization to achieve a high gain and a wide impedance bandwidth, validated through fabrication and testing.

Zhao et al. [27] explored a five-degree-of-freedom electromagnetic levitation actuator for laser cutting machines. Their work offers a compelling solution for achieving high-speed, high-precision control, which is crucial for advanced manufacturing. They employed nonlinear analytical modeling, finite element simulations, and PID-based centralized control to achieve high-speed and high-precision lens manipulation in laser cutting applications.

Huang et al. [28] proposed a high-efficiency 2.45 GHz rectifying circuit for RF energy collection systems. This innovative approach could pave the way for sustainable energy solutions in IoT and low-power devices. Their approach focused on suppressing harmonic components and optimizing the rectifier’s DC-RF conversion efficiency through detailed simulation and experimental comparisons.

Ahmed et al. [29] and Abdou et al. [30] introduced compact and high-performance devices for 5G and beyond. Ahmed et al. focus on a quasi-twisted branch-line coupler, while Abdou et al. present a multiband millimeter-wave dielectric resonator antenna with omnidirectional radiation capabilities. By utilizing a double-layered microstrip line structure with a slow-wave design, Ahmed et al. achieved a significant size reduction and enhanced bandwidth, supported by their simulation and fabrication results. Abdou et al. utilized the excitation of specific electromagnetic modes and validated their design through simulation and measurements.

Behera et al. [31] investigated circularly polarized metasurface antennas tailored for hybrid wireless applications. This study emphasizes energy-efficient designs that cater to IoT and smart sensor networks. Behera et al. adopted AI-driven surrogate model-assisted optimization to design a polarization-reconfigurable metasurface antenna. Their study combined the use of smart metasurfaces with reconfigurable monopole antennas to achieve high gain and broad bandwidth.

Li et al. [32] contributed a novel dual-polarized patch antenna with enhanced isolation, showcasing its utility in modern wireless communication systems, where signal clarity and separation are paramount. Their design methodology incorporated equivalent circuit modeling and rigorous design formulas to enhance isolation and bandwidth.

Hu et al. [33] examined the compatibility of flexible UHF antenna sensors with SF6/N2 gas mixtures. Their findings support the development of reliable sensors for high-voltage applications. Their experimental approach combined Fourier-transform infrared spectroscopy, scanning electron microscopy, and X-ray photoelectron spectroscopy to analyze material interactions.

Zhan et al. [34] proposed a Ka-band MEMS delay with low insertion loss and high accuracy. This design has significant implications for phased-array radar and communication systems. Their method involved optimizing the structure to minimize insertion loss and enhance delay accuracy through system simulations.

Neto et al. [35] explored the development, applications, and potential of planar printed structures inspired by Matryoshka geometries. These structures leverage the nesting principles of Matryoshka dolls to achieve compact, multi-resonance, and wideband configurations. This study demonstrates various applications of planar printed circuit technology, focusing on frequency-selective surfaces (FSSs), filters, antennas, and sensors.

Wang et al. [36] introduced a terahertz metamaterial absorber based on vanadium dioxide (VO_2_) that achieves switchable ultra-wideband and ultra-narrowband absorption by leveraging the material’s phase-transition properties. The proposed absorber comprises a multilayer structure with VO_2_ as the topmost layer, supported by an insulating Topas layer, a PMI dielectric layer, and a gold reflector.

Weng et al. [37] proposed a miniaturized loaded open-boundary quad-ridge horn (LOQRH) antenna engineered for interferometric direction-finding systems. By optimizing the ridge structure and incorporating resistive loading and a self-balanced feed, they effectively suppressed common-mode currents, ensuring radiation pattern symmetry and minimizing phase center fluctuations. This work underscores the potential of compact LOQRH antennas to improve accuracy and efficiency in multiprobe interferometric applications.

In conclusion, this Special Issue focused on the potential of recent progress in electromagnetic devices to address critical challenges across various applications. The papers included in this Special Issue demonstrate the diversity and impact of cutting-edge research in this field, from advanced wireless communication systems and energy-harvesting technologies to precision manufacturing and high-performance antennas. A key theme emerging from these works is the emphasis on innovative design methods and multidisciplinary approaches. For example, the development of reconfigurable metasurfaces, compact and high-efficiency components for 5G and beyond, and advanced actuators for laser machining illustrates the continuous push for solutions that combine functionality, precision, and scalability. Similarly, efforts to enhance energy-harvesting efficiency, improve antenna isolation, and enable omnidirectional radiation patterns reflect the field’s commitment to addressing practical needs in real-world applications. Another notable aspect is the range of applications explored, from 6G communications, radar sensing, and hybrid wireless systems to high-voltage power monitoring and phased-array systems.
